# Type VI secretion system: secretion by a contractile nanomachine

**DOI:** 10.1098/rstb.2015.0021

**Published:** 2015-10-05

**Authors:** Marek Basler

**Affiliations:** Focal Area Infection Biology, Biozentrum, University of Basel, Basel, Switzerland

**Keywords:** type VI secretion system, contractile phage tail, dynamics, structure, effectors, energetics

## Abstract

The type VI secretion systems (T6SS) are present in about a quarter of all Gram-negative bacteria. Several key components of T6SS are evolutionarily related to components of contractile nanomachines such as phages and R-type pyocins. The T6SS assembly is initiated by formation of a membrane complex that binds a phage-like baseplate with a sharp spike, and this is followed by polymerization of a long rigid inner tube and an outer contractile sheath. Effectors are preloaded onto the spike or into the tube during the assembly by various mechanisms. Contraction of the sheath releases an unprecedented amount of energy, which is used to thrust the spike and tube with the associated effectors out of the effector cell and across membranes of both bacterial and eukaryotic target cells. Subunits of the contracted sheath are recycled by T6SS-specific unfoldase to allow for a new round of assembly. Live-cell imaging has shown that the assembly is highly dynamic and its subcellular localization is in certain bacteria regulated with a remarkable precision. Through the action of effectors, T6SS has mainly been shown to contribute to pathogenicity and competition between bacteria. This review summarizes the knowledge that has contributed to our current understanding of T6SS mode of action.

## 1. Discovery of novel secretion system

Gram-negative bacteria use various secretion systems to deliver proteins from the bacterial cytosol to the extracellular space or into target cells, and quite often these systems are important virulence factors [1]. Indeed, the type VI secretion system (T6SS) was discovered when Pukatzki *et al.* [2] used *Dictyostelium discoideum* as a model organism to screen many isolates of *Vibrio* *cholerae* for novel virulence factors. The screen identified non-O1, non-O139 *V. cholerae* strain V52 that uses a conserved cluster of genes to resist a predation by amoebae. Similar clusters of genes were previously identified as conserved in many other Gram-negative bacteria but their function was not known [3]. Importantly, Pukatzki *et al.* showed that the gene cluster is responsible for secretion of haemolysin-corregulated protein (Hcp), which was previously identified as secreted by an unknown mechanism [4], and three VgrG proteins one of which was previously shown to contain toxic actin cross-linking domain [5]. Shortly after characterization of T6SS in *V. cholerae*, one of the three T6SS clusters of *Pseudomonas* *aeruginosa* was shown to secrete Hcp *in vitro* and because the Hcp was also detected in the sputum of cystic fibrosis patients infected by *P. aeruginosa* it was suggested that this system could be important for pathogenesis [6].

Transport of proteins across a barrier needs a source of energy and accordingly the early analyses of T6SS cluster components identified two putative ATPases, TssM (IcmF) and ClpV (TssH). Full-length TssM protein was shown to be important for T6SS function in *V. cholerae* [2]; however, the early observation that the ATPase activity of TssM is dispensable for T6SS activity in *Edwardsiella* *tarda* [7] indicated that ClpV could be the essential ATPase powering the T6SS and TssM could play an important structural role. Indeed, Mougous *et al.* [6] showed that localization of ClpV, detected by fluorescence microscopy, correlates with T6SS activity and that its ATPase activity is necessary for Hcp secretion. ClpV is similar to other AAA (ATPases Associated with diverse cellular Activities) proteins, like ClpB, and its ATPase activity was previously also correlated with the ability of *Salmonella typhimurium* and *Yersinia pseudotuberculosis* cells to enter epithelial cells [8]. Since ClpB and homologous AAA proteins are known to unfold and thread substrates through their pore, it seemed reasonable that ClpV could be involved in pushing T6SS substrates across the cell membranes. Interestingly, Hcp crystal structure suggested that stacks of Hcp hexamers could form a channel for T6SS substrates [6].

Overall, these initial studies clearly showed that a conserved gene cluster was responsible for protein secretion and virulence by a mechanism distinct from previously described secretion systems [2,6]. For details of the work that predated the discovery of T6SS, see the reviews by Filloux *et al*. [9] and Bingle *et al*. [10].

Discovery of T6SS triggered a wide range of follow up research focused on answering the basic questions such as: What is the molecular mechanism of type VI secretion? How is T6SS regulated? What are the effectors that are secreted by various organisms and what is their mode of action? How important is T6SS during pathogenesis or in the environment? Over a decade of research led by many laboratories hugely improved our understanding of T6SS. Many comprehensive reviews about different aspects of T6SS were written recently, either with a broad view [11–13], or with a focus on effectors [14,15] or on structural aspects [1,16–18]. Here, I will review the progress that has been made towards understanding the molecular mechanism of protein secretion by T6SS and discuss its unique mode of action.

## 2. Towards an ‘inverted phage tail’ model of T6SS function

A model of T6SS mode of action changed fundamentally after the discovery that many critical components of T6SS are structurally and thus also potentially functionally homologous to components of contractile phage tails. First, secreted VgrG proteins were shown to be structural homologues of T4 phage spike complex gp5–gp27 [19,20]. Hcp protein was shown to be a structural homologue of a phage tube protein [19,21]. Moreover, structural modelling and predictions suggested that gp25, a conserved component of T4 phage baseplate, is homologous to an essential T6SS protein, TssE [10,19,22]. Surprisingly, Bönemann *et al.* [23] showed that the substrate of ClpV was a cytosolic protein VipB (TssC) and not the secreted proteins Hcp and VgrG. The authors also nicely showed that VipA (TssB) and VipB proteins assembled into a tubular polymer that can be disassembled by ClpV *in vitro* [23]. Even though the biological significance of this tubular structure was not immediately clear, Leiman *et al.* [19] noted that its overall structure resembled T4 phage polysheath. Overall, these observations suggested that T6SS could function as an inverted phage tail and use the contraction of a sheath-like structure to drive the Hcp tube with associated VgrG-effector spike out of the cells [19,24,25].

**Figure 1.**
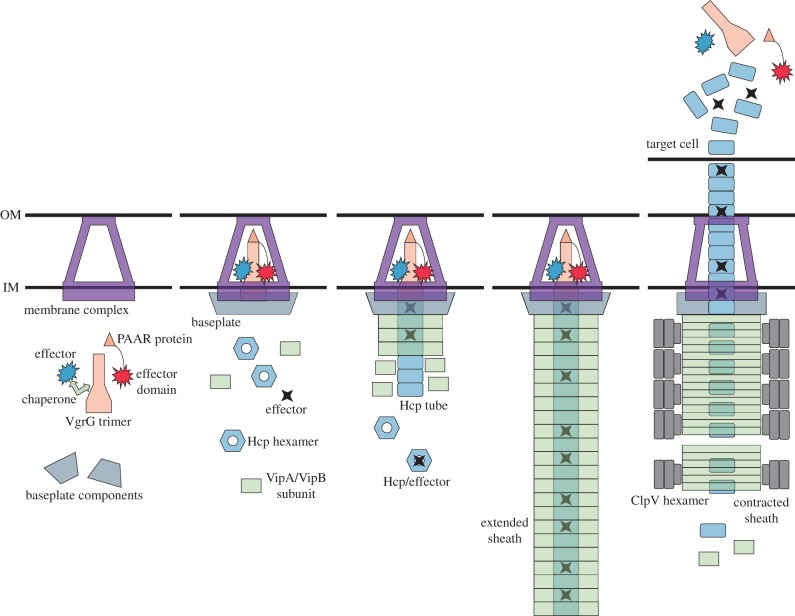
T6SS mode of action. Assembly of T6SS in an extended ‘ready to fire’ conformation starts by the assembly of a membrane complex composed of TssJLM. Effector domains can be either present on VgrG C-terminus and PAAR C- and N-termini or be preloaded onto VgrG/PAAR spike complex, optionally with an assistance of non-secreted chaperones. VgrG/PAAR/effector complex possibly together with TssEFG and K proteins form the baseplate in a conformation that initiates assembly of tube and sheath. Hexameric rings of Hcp, potentially with bound effectors, assemble to a long rigid tube that serves as a template for the assembly of an extended VipA/VipB sheath. Potential conformational change in the baseplate/membrane complex triggers the sheath contraction, which pushes the Hcp tube with the VgrG/PAAR spike and associated effectors from the cell to an extracellular space or across a target cell membrane. The contracted sheath is specifically recognized by ClpV ATPase, which unfolds the subunits and thus recycles them for a new round of assembly of an extended sheath.

The first idea about overall structure and mode of action of T6SS came from a study of T6SS in *V. cholerae* [26]. This study was possible due to an enormous progress in electron microscopy (EM) of bacterial ultrastructures in three dimensions in their native state inside intact cells [27,28]. Whole cell cryo-electron tomography showed that T6SS indeed resembles a long phage tail attached to the cell envelope by a membrane anchor. The tail was visualized in two conformations, extended and contracted, which resembled the previously identified VipA/VipB sheath [23,26]. The contracted sheath structures were in general shorter, wider and apparently empty as opposed to the extended structure that had an extra density inside, which was suggested to be an Hcp tube [26]. The realization that the extended structures span the whole bacterial cytosol initiated fluorescence microscopy analysis of dynamics and subcellular localization of T6SS assembly by imaging of VipA-GFP in live cells. It was shown that T6SS sheath assembly in *V. cholerae* takes about 20–30 s, then the sheath contracts to about half its length in less than 5 ms and the contracted sheath is disassembled over tens of seconds in a ClpV-dependent manner [26]. Since then, the T6SS dynamics was described using live-cell imaging in more detail in *V. cholerae*, *P. aeruginosa* and *Escherichia* *coli* [29–31]. In summary, the description of an overall structure and fast dynamics of the assembly showed that T6SS has a fundamentally different mode of action from that of other known secretion systems (figure 1).

## 3. Initiation and regulation of T6SS assembly

Live-cell fluorescence microscopy showed that the T6SS sheath does not assemble in cells lacking critical T6SS components [26,31], suggesting that the membrane anchoring complex and a baseplate are necessary for initiation of tube and sheath polymerization.

Recently, great progress has been made towards understanding of T6SS tail attachment to a bacterial cell envelope. The minimal membrane complex is formed by the inner membrane proteins TssL and TssM, which are homologues of type IV secretion system components IcmF and DotU, and an outer membrane lipoprotein TssJ [32–37]. TssM is anchored into an inner membrane by three transmembrane segments and interacts with TssL [36]. Furthermore, TssJ was shown to form a complex with TssM and TssL [33,35]. The OmpA-like extension domain of TssL, or in some organisms like *E. coli* an additional accessory protein TagL, anchors the membrane complex to the peptidoglycan [38]. Structures of TssL from *Francisella novicida*, *V. cholerae* and *E. coli* [39–41], of TssJ from *P. aeruginosa*, *E. coli* and *Serratia* *marcescens* [35,42,43] and partial structure of *E. coli* TssM C-terminal domain alone or in a complex with TssJ were solved, providing a very detailed picture of the membrane complex assembly [34].

Importantly, the whole TssJLM complex of *E. coli* was recently isolated and resolved at 12 Å resolution by EM, providing an unprecedented insight into the overall assembly [34]. The complex has fivefold symmetry and is composed of 10 copies of each component (TssM, L, J) with an overall mass of 1.7 MDa. The complex forms a 30 nm high and 20 nm wide rocket shaped structure that spans both inner and outer membranes with only a narrow pore. The complex was proposed to undergo conformational changes to accommodate the spike with effectors and Hcp tube passing through [34]. Importantly, functional N-terminal fusions of sfGFP to TssM and TssL localize into one or two static and stable foci on the cell periphery and the T6SS sheath polymerizes from these stable complexes repeatedly. Furthermore, TssJ was shown to be the nucleation factor for the membrane complex assembly [34].

Functions of TssE, TssF, TssG, TssK and TssA proteins are currently not well understood. These proteins could be involved in formation of a baseplate connecting the T6SS tube and sheath to the membrane complex (figure 1). Indeed, in *E*. *coli*, TssK was shown to interact with the membrane complex protein TssL as well as with Hcp and VipB components of the sheath [44] and, in *S. marcescens*, TssK was shown to interact with TssF and TssG [45]. Because TssE and its phage homologues have a fold that is similar to the fold of the inner domain of T6SS or phage sheath, it was suggested that TssE could be directly binding the sheath [24,46,47].

As reviewed recently, the expression of T6SS gene clusters is regulated by various environmental clues and many organisms even have multiple independently regulated T6SS with different functions [17,48,49]. However, from a structural point of view, it is interesting that the T6SS assembly is regulated also post-translationally in some organisms. For example, in the first of the three T6SS clusters of *P. aeruginosa* (H1-T6SS), the accessory protein TagH is phosphorylated by a cognate serine–threonine kinase, PpkA, and dephosphorylated by a phosphatase, PppA [50]. TagH is also phosphorylated in *S. marcescens* [51] but in *Agrobacterium tumefaciens* T6SS assembly is regulated by phosphorylation of TssL [52,53]. In H1-T6SS of *P. aeruginosa*, PpkA activation requires a periplasmic protein, TagR [54], which is anchored to the outer membrane by an interaction with TagQ [55]. Additionally, TagT and TagS were shown to form an inner membrane complex with ATPase activity and act upstream of PpkA [55]. Interestingly, in *P. aeruginosa*, the H1-T6SS can be also activated independently of TagH phosphorylation by inactivation of another accessory protein, TagF [56].

Live-cell imaging of H1-T6SS dynamics in *P. aeruginosa* showed that the cells are able to initiate and subcellularly localize the assembly of their T6SS in a response to T6SS activity of a neighbouring sister cell with a remarkable spatial and temporal precision [29]. Interestingly, it has been also shown that *P. aeruginosa* kills T6SS+ organisms such as *V. cholerae*, *Acinetobacter baylyi* or *Burkholderia thailandensis* better than their T6SS− mutants [57,58]. This phenotype called ‘duelling’ is regulated by the TagQRST/PpkA signalling cascade [57], which can also respond to mating-pair formation initiated by T4SS or to membrane damage induced by polymyxin B [59]. Importantly, the spatio-temporal regulation of the T6SS assembly allows *P. aeruginosa* to preferentially attack T6SS+ cells even in a mixture with T6SS− cells and this is independent of the level of T6SS expression in *P. aeruginosa* [57,59]. However, regulation of expression level of H1-T6SS in response to lysis of kin cells further contributes to an efficient use of T6SS in *P. aeruginosa* [60].

Overall, these studies suggest that a proper assembly and structural changes of the membrane complex and baseplate can be used for an efficient spatio-temporal regulation of T6SS activity. This has an analogy in phages where the assembly of a baseplate is required for tube and sheath polymerization [25,61]. Interestingly, a change of the structure of the phage baseplate initiates contraction of the sheath upon binding to the host cell [25,62,63]. This suggests that contraction of the T6SS sheath may also be triggered by structural changes in the baseplate and possibly also in the membrane complex (figure 1).

## 4. Assembly of the tube and sheath

In phages, the fully assembled baseplate includes also the spike complex, which initiates the assembly of a tail tube and an extended sheath around it [24,64–66]. The integrity of VgrG/PAAR spike complex was indeed shown to be essential for T6SS function [2,31,67], and even though the Hcp tube assembly has not been directly visualized yet, it has been shown that Hcp tube and sheath polymerize by a mechanism similar to phage [68].

Even before realization that Hcp is a structural homologue of phage tube protein [19,21], it was proposed that Hcp could form a conduit (channel) for T6SS effectors because Hcp of *P. aeruginosa* was shown to form hexameric rings that were stacked into a tube in a crystal lattice [6]. Indeed, introducing cysteines to a surface between Hcp rings can lead to cross-linking of Hcp into a tube *in vitro* [69,70]. Similarly, in *E. coli*, by positioning cysteine on Hcp to probe various possible assemblies of Hcp rings, it was shown that Hcp rings assemble head-to-tail *in vivo* and that this assembly is dependent on the presence of T6SS components essential for tail assembly [68]. It is important to note that in these studies cysteine cross-links were designed for Hcp rings stacked one on top of another (head to tail) without any helical twist [68–70].

T4 phage extended sheath assembly is kinetically driven by interaction of sheath monomers with tube template [64,71]. In T6SS, Hcp was shown to interact with the VipA component of the T6SS sheath using a bacterial two hybrid system [68] and the extended sheath does not assemble in the absence of Hcp [31]. N-terminal negatively charged and C-terminal positively charged residues on Hcp were shown to be important for Hcp secretion in *E. tarda* [72]. Since these residues are on the surface of the Hcp ring, they could be involved in Hcp–sheath interaction. These experiments suggest that in an extended conformation Hcp rings interact with sheath subunits through charge interactions similarly to interactions described recently at the atomic level for R-type pyocin [46]. Importantly, an atomic model of the T6SS sheath shows that its inner layer has the same fold as the phage sheath, suggesting that the tube–sheath interaction and the mechanism of assembly is conserved between phage and T6SS [47]. Interestingly, in the fully assembled extended tail of R-type pyocin, the inner tube has the same helical parameters as the outer sheath [46]. It is not known if the length of the T6SS tail is regulated and whether the assembly is terminated by a cap.

## 5. Powering the secretion by sheath contraction

The contraction of a sheath powers the secretion of effectors and also puncturing of a target cell membrane by T6SS, phage or R-type pyocin. A mechanism of T4 phage sheath contraction was first proposed based on the EM analysis of extended, contracted and partially contracted T4 tails [73,74] and further improved based on cryoEM analysis and partial atomic structure of the gp18 sheath subunit [62,63,75]. Recently, an atomic model of an R-type pyocin particle was solved in both extended and contracted states, and thus can serve as a model for estimation of energy released during a contraction [46]. During contraction, rings of sheath collapse sequentially to form a more compact structure that is stabilized by newly formed charge interactions [46]. Energy gained during the contraction of a pyocin sheath was estimated to be 12 kcal mol^−1^ per subunit [46], which is about half of what was previously measured for the twice as large T4 sheath [65].

To estimate how much energy is released during the contraction of the T6SS sheath, atomic models of both states, including details of Hcp–sheath interaction in the extended conformation, would be necessary. So far, only atomic models for contracted states of sheath of *V. cholerae* and *Francisella tularensis* are available [47,76]. Analysis of these atomic models showed that the T6SS sheath is composed of three domains. T6SS-specific domain 3 is on the surface of the sheath and plays a role in the recycling by ClpV (see below). In the inner two layers of T6SS sheath, subunits have the same fold as the whole pyocin sheath subunit and individual subunits are interconnected by a similar mesh of augmented β-strands in the inner layer of the sheath polymer. This suggests that a similar amount of energy could be released during a contraction of pyocin and T6SS sheath [46,47,76].

Assuming that the energy gain per subunit is at least as big for T6SS as it is for the R-type pyocin and that a 1 µm long T6SS sheath is composed of approx. 1500 sheath subunits, the total energy gain from a single contraction could be 18 000 kcal mol^−1^. Energy that is released by hydrolysis of one ATP molecule is approx. 11.2 kcal mol^−1^ depending on growth conditions [77]. Therefore, one contraction event would release energy equivalent to hydrolysis of 1600 molecules of ATP. Since the contraction happens in less than 5 ms, to get a similar power output would require an equivalent of at least 32 000 molecules of ClpX, which has a maximum ATPase rate of 10 s^−1^ [78]. For comparison, a syringe-like injection mechanism of Tc toxins of *Photorhabdus luminescens* was described at the atomic level and it was predicted that during a transition of TcA from pre-pore to pore about 20–66 kcal mol^−1^ of energy is released [79,80].

The speed of substrate translocation is remarkable as well. Since a sheath contracts to about 50% of its original length, an average sheath of length of 1 µm moves the VgrG/PAAR/effector payload at a speed of at least 100 µm s^−1^. For comparison, kinesin moves at a rate of around 0.5 µm s^−1^, depending on the load, and consumes about one ATP molecule per 8 nm [81]. Moreover, in a phage and R-type pyocin, a tip of a tube rotates as it leaves a baseplate [46,62]. Since it is approximately one turn per 100 nm for T4 phage tail [62], in the case of T6SS we could predict up to 10 turns in less than 5 ms, in other words up to 120 000 revolutions per minute.

Overall, it is quite clear that the amount of energy that is released during T6SS sheath contraction and the speed at which a payload moves and rotates is quite remarkable, suggesting that T6SS has the potential to ‘drill’ large cargo across membranes.

## 6. Recycling of T6SS sheath by ClpV

It is important to realize that at least in *V. cholerae*, sheath unfolding by ClpV ATPase is not essential for T6SS activity. It was shown that cells lacking ClpV are still capable of killing *E. coli* in a T6SS-dependent manner and assemble similar sheath structures as wild-type cells [26,82]. This is very likely due to rapid cell growth and de novo synthesis of sheath components because contracted sheath was not observed to extend again or to disintegrate to individual subunits [26]. However, to allow for successive rounds of assembly of T6SS tail in an extended conformation, the contracted sheath has to be actively disassembled by ClpV into individual subunits [23,29]. Moreover, ClpV also disassembles contracted sheaths that can form from soluble subunits even in the absence of functional T6SS, and thus increases the concentration of soluble subunits for efficient sheath assembly [23,31].

ClpV has N-terminal and two AAA domains separated by a middle domain and its primary sequence is about 35% identical to ClpB, which is involved in a disassembly of large protein aggregates [8]. The N-terminal domain of ClpV, composed of eight alpha helices, is structurally similar to those of ClpA/B/C but contains a ClpV-specific N-terminal *α*-helix involved in binding of VipB [83]. Co-crystal structure shows that residues 15–28 of VipB are recognized by a hydrophobic groove of the N-terminal domain of ClpV [83]. Point mutations in the N-terminal domain of ClpV block binding of ClpV to contracted sheath *in vitro* [83] and *in vivo* [29].

ClpV of *V. cholerae* forms hexameric rings in the presence of ATP and those rings have higher affinity to VipA/VipB; moreover, ClpV also binds preferentially to a polymeric VipA/VipB structure [23,83]. After binding of VipA/VipB, the VipB N-terminus is threaded through the pore of ClpV while ATP is hydrolysed. Mutations in the pore were shown to block sheath disassembly *in vitro* [23,83], and since the binding to the substrate is not blocked, the mutant ClpV colocalizes with contracted sheaths in live cells [29]. It is however not clear whether the full-length VipB is threaded through the ClpV or if pulling on VipB leads to destabilization of the whole VipA/VipB polymer and disassembly to subunits. The amount of unfolding and length of the substrate has consequences for energy that is needed for recycling of the contracted sheath.

Preferential binding of ClpV to polymeric structures suggests that ClpV is not constantly refolding VipA/VipB sheath subunits; however, since extended and contracted sheaths are composed of the same subunits, ClpV has to specifically recognize only the contracted sheaths to allow for extended sheath assembly. Indeed, high-speed live-cell imaging of localization of both VipA and ClpV in *V. cholerae* revealed that ClpV is dispersed in the cytosol in the presence of an extended sheath; however, immediately after a contraction it relocalizes to the contracted sheath [29]. After only few seconds, a maximum binding between ClpV and the contracted sheath is reached and the sheath starts to fall apart to smaller pieces in the next tens of seconds [29]. This suggests that the surface of the sheath changes during its contraction (figure 1).

The easiest explanation is that the N-terminal *α*-helix of VipB is not accessible to ClpV on an extended sheath but is exposed on the sheath surface after its contraction. Indeed, recent medium-resolution structure [84] and also atomic models of the contracted sheaths of *V. cholerae* and *F. novicida* [47,76] provided the first data to support such a mechanism. These structures clearly show that the T6SS sheath is composed of three layers: the inner two layers are highly homologous to the phage and R-type pyocin sheaths and the surface layer is specific to T6SS sheaths. Importantly, the surface exposed part of domain 3 is composed of three N-terminal *α*-helices of VipB and two C-terminal *α*-helices of VipA [47,76]. An extended T6SS sheath was modelled based on an extended T4 phage sheath and this showed that domain 3 is likely to be hidden between sheath strands on the extended sheath and thus potentially inaccessible to ClpV binding [47,84]. It is important to realize, however, that the exact localization and structure of the N-terminus of VipB is unknown because it was not resolved at a high enough resolution [47,76,84]. Alternatively, new interactions that are established on the contracted sheath may induce destabilization of interactions between *α*-helices in domain 3 and thus expose the N-terminus of VipB for ClpV binding. Clearly, atomic models of T6SS tail in both extended and contracted states and biophysical measurements are necessary to fully understand the molecular mechanism of sheath disassembly.

In some T6SS, such as H1-T6SS of *P. aeruginosa*, sheath recycling is apparently more complicated. The structure of the N-terminal domain of *P. aeruginosa* ClpV was found to be different from that of *V. cholerae* ClpV. This change blocks binding of the N-terminus of VipB by the ClpV N-terminal domain suggesting a different mechanism of sheath recycling [85]. Interestingly, an accessory protein HsiE1/TagJ, not essential for T6SS function, was shown to bind to both the VipA N-terminus and ClpV [85,86]. *V. cholerae* VipA N-terminus seems to be buried in domain 2 of a sheath and thus is probably accessible only from the end of the T6SS sheath but unlikely from the sheath surface [47,84]. It was therefore suggested that ClpV–VipB interaction, different from ClpV–VipB interaction in *V. cholerae*, leads to an initial fragmentation of a contracted sheath and thus exposure of free ends of the contracted sheaths, which in turn increases subsequent recruitment of TagJ/ClpV for a complete disassembly [85]. This mechanism could make the whole recycling process more efficient because VipA is a smaller protein and its unfolding might require less ATP. The process could be faster because of simultaneous disassembly from ends and sides of sheath fragments. This mechanism may be also important for preventing aggregation of sheath monomers similarly to what was shown for ClpV in *V. cholerae* [31]. It will be interesting to learn how disassembly of an assembled extended sheath is prevented from the exposed end.

## 7. Potential costs of T6SS secretion

Contracted sheath has its VipA/VipB rings about 2.1 nm apart [47,84] and is formed from an extended sheath that is about twice as long and usually stretches across the whole width of a cell [26]. Therefore assuming a 1 µm long extended sheath, the full width of an average sized bacterial cell, one sheath structure is composed of approximately 250 rings and thus about 1500 subunits of VipA and VipB. Since ClpV probably works similarly to other AAA unfoldases, the cost for remodelling of sheath could probably be separated into two parts: (i) the cost of denaturation of sheath subunits and (ii) the cost of translocation of the unfolded VipB protein through the ClpV pore. The cost of denaturation varies hugely based on local stability of the protein that is being unfolded and can be as little as a few ATP molecules, up to hundreds of ATP molecules [78]. To minimize this cost, the VipA/VipB subunit could be optimized for cooperative unfolding and low stability of the region next the VipB N-terminus. The cost of polypeptide translocation through the pore depends on the length of a substrate and was estimated to be approximately one ATP per amino acid in the case of ClpXP translocating a denatured substrate [78]. If indeed full-length VipB protein were to be threaded through ClpV, up to 500 molecules of ATP could be consumed per subunit and that could be as much as 750 000 molecules of ATP for a single sheath. However, it is possible that it is not necessary to unfold the full-length VipB to disassemble a contracted sheath. As ClpV pulls on the VipB N-terminus, the sheath polymer could be destabilized and broken into individual subunits. The unfolding of VipB could be then terminated by dissociation of ClpV from the monomeric sheath subunit, thus saving ATP.

Another important cost comes from the fact that Hcp is a structural component, which is secreted into the environment in large quantities in many T6SS+ organisms. Hcp can be detected as an abundant secreted protein in organisms such as *V. cholerae*, *A. baylyi*, *S. marcescens*, *P. aeruginosa* and *E. tarda* [2,6,7,67,87]. Because of the extensive structural and functional homology between T6SS and contractile tails, it is probably safe to assume that each ring of sheath interacts with one ring of Hcp as it is in the R-type pyocin [46]. This means that up to 700 molecules of Hcp are secreted out of the cell every time a full-length sheath contracts. Assuming only one contraction per 5 min and the molecular weight of Hcp to be 18 kDa, then 10^9^ cells (1 ml of OD 1 culture) will secrete about 250 ng of Hcp into the supernatant in 1 h. This seems to be a huge waste especially considering that only three molecules of VgrG and one molecule of PAAR protein are secreted during each secretion cycle [19,67]. As explained below, the T6SS spike can be decorated by many different effectors at the same time and some effectors bind into the lumen of the Hcp ring [88] thus potentially increasing the overall efficiency of T6SS.

## 8. Discovery of T6SS effectors

Many T6SS effectors were identified using mass spectrometry analysis of supernatants of T6SS+ organisms. This led to the initial discovery of VgrG in *V. cholerae* [2], Tse effectors in *P. aeruginosa* [89], and many effectors of *B. thailandensis* [90], *S. marcescens* [51] or *V. parahaemolyticus* [91]. Even though this is a very straightforward and unbiased approach, it has its own limitations. The biggest problem is that certain classes of effectors, such as VgrG-associated proteins, are secreted at a very low rate and thus the protein abundance in the supernatant could be below the detection limit. Another problem could be that some T6SS systems are tightly regulated and trigger secretion in a response to an attack or environmental clues [57–60].

An elegant approach to identify antibacterial effectors was developed by Dong *et al.* [92] The principle of this method is that T6SS+ organisms that secrete antibacterial effectors use specific immunity proteins to block their action during growth in close contact with sister cells. If an immunity gene is disrupted by transposon mutagenesis, such a mutant will be killed by its T6SS+ neighbours. Viability of mutants in the presence or absence of active T6SS is then scored using deep sequencing. Mapping these T6SS-dependent immunity proteins helps to identify cognate effectors that are usually located in the same operon [92]. This approach is, however, limited to effectors that have exactly one cognate immunity protein targeted into the periplasm of the effector cell.

Another interesting mass spectrometry method was based on the fact that some Hcp-binding effectors are less stable in the absence of Hcp [88,93]. An advantage of this method is that proteins that are active in the cytosol or are not necessarily toxic against bacteria can be detected, but only in the case of their inherent instability in the absence of Hcp [93].

Bioinformatics has proved to be a useful approach for finding new effectors for various secretion systems including T6SS [94]. Some T6SS effectors are part of characteristic gene clusters or have certain physical properties such as pI or size, which facilitates their identification [90]. Furthermore, certain conserved domains are found present in T6SS effectors, such as PAAR [67,95] or the MIX domain first found in *V. parahaemolyticus* [91]. It is also now clear that many effectors are located in the operons together with their cognate VgrG, Hcp or PAAR proteins [88,96,97].

## 9. Mechanism of effector secretion

We can conceptually divide T6SS effectors based on various criteria. For example, based on a target, we can divide effectors into antibacterial, anti-eukaryotic or being active against both targets as shown recently [98,99]. Not all T6SS substrates need to be necessarily toxic to target cells. In *Y. pseudotuberculosis*, a T6SS substrate YezP was shown to bind to Zn^2+^ and increase its acquisition, and thus helps the bacteria to survive stress and host immunity during pathogenesis [100]. We can also characterize the effectors based on their structure and mechanism of secretion. Here, I will only briefly discuss the mechanisms of effector secretion. For very comprehensive recent reviews of T6SS effectors and their function refer to Durand *et al*. [14] and Russell *et al*. [15].

The current model of how effectors are secreted out of the cells is based on what we know about the overall structure and dynamics of T6SS and also based on the structural and functional homology to contractile phage tails (figure 1). VgrGs were the first class of proteins shown to be secreted out of T6SS+ cells [2]. VgrGs are structural homologues of the phage spike complex and even though VgrG–Hcp interaction was only shown in *A. tumefaciens* [52], it is believed that VgrG forms a trimer at the very end of an Hcp tube [19,20]. The VgrG N-terminal domain forms a pseudo-dimer that has a fold similar to Hcp dimer, and therefore VgrG trimer docks nicely to Hcp hexamer [19].

The first discovered T6SS effector was so-called ‘evolved’ VgrG protein, with VgrG N-terminal domain and actin cross-linking effector domain at the C-terminus [20]. Since then, more VgrG fusion effectors were characterized, such as membrane fusion mediating VgrG-5 of *B. pseudomallei* [101,102], actin ADP-ribosylating VgrG1 of *Aeromonas hydrophila* [103], or peptidoglycan hydrolysing VgrG-3 of *V. cholerae* [92,104]. Secretion of several effectors is dependent on a particular VgrG, like TseL of *V. cholerae* or Tse5 and Tse6 of *P. aeruginosa* [92,93]. Interestingly, chaperone proteins that load effectors onto their cognate VgrGs but are not secreted were discovered recently in *V. cholerae* [105,106]. Searching for these conserved proteins led also to identification of genetically linked effectors in *A. hydrophila* [105]. This mechanism could provide another level of regulation since expression of different chaperone proteins could lead to loading of different combinations of effectors onto a spike.

VgrG trimer is sharpened by a small PAAR protein, which is a structural component essential for T6SS function. Similarly to VgrG, PAAR proteins may bind or be fused to effectors [67]. For example, large Rhs-domain containing T6SS effectors of *S. marcescens*, *P. aeruginosa* or *Proteus mirabilis* contain a PAAR domain [93,107–109]. Importantly, a fully assembled VgrG/PAAR spike is needed for proper T6SS function and Hcp secretion [2,20,67]. Moreover, it seems that deletion of several spike-associated effectors can also lead to a decrease of T6SS activity [92]. This suggests that a certain mechanism controls full assembly to prevent wasteful secretion of a spike that lacks effectors.

Many structures of Hcp proteins are available and all Hcp proteins form a hexameric ring with an inner diameter of approximately 4 nm [6,70,72,110,111]. Interestingly, Hcp was shown to have a chaperone-like activity and bind small effectors in *A. tumefaciens*, *E. tarda* and *P. aeruginosa* [7,52,88,112]. Moreover, examples of Hcp with extension domains were found as well [113]. Overall, it is becoming clear that the effectors are loaded onto the extended T6SS during the assembly as a fusion to or by an interaction with the secreted structural components and then secreted all at once together with the Hcp/VgrG/PAAR protein complex [67].

The example of *P. aeruginosa* H1-T6SS shows that different substrates can be secreted by distinct mechanisms from the same organisms [93]. It is, however, not clear whether the same T6SS machine can indeed deliver multiple effectors in a single contraction event or if multiple assemblies are required. It is also not clear whether the Hcp tube is fully loaded with its interacting effectors or if binding of effectors is only sparse. Another unresolved question is where exactly the effectors are located on the VgrG/PAAR spike and how large a cargo can be loaded at once. Clearly, a sharp tip evolved to facilitate membrane puncturing in both T6SS and phages [67,114]. Therefore, it seems unlikely that effectors are directly on the tip of VgrG/PAAR and are more likely on the side of the VgrG as is lysozyme on gp5 of the T4 phage spike [115].

Mechanical puncturing of a membrane to deliver large folded hydrophilic effectors would be necessary and thus there are probably certain physical limits to how large effectors can be. On the other hand, Rhs are known to form a large beta-stranded cage that encapsulates toxic molecules in an unfolded state and can open to release then upon a signal [80,116], and thus the fact that Rhs can be substrate of T6SS shows that the size limits might be relatively high.

Interestingly, antibacterial effectors with both periplasmic and cytosolic targets are secreted by T6SS [89,93,96]. Even though Hcp tubes are presumably long enough to reach the target cell cytosol [26], it is not clear how far into the bacterial cell can the Hcp/VgrG/PAAR with the associated effectors be delivered. There are several options: (i) all effectors are delivered into the periplasm of the target cell and use separate mechanisms to cross the inner membrane, (ii) effectors are delivered into the cytosol and then some are transported to the periplasm, or (iii) physical properties of an effector dictate in which compartment it dissociates from the tube or spike.

R-type pyocins of *P. aeruginosa* or *Clostridium difficile* can punch a hole into a bacterial cell by a contraction of its tail [117–119]. The stable inner tube that remains inserted in the cell envelope creates a conduit for ions and ion leakage from the bacterial cell then leads to the cell death [46]. This mechanism is so potent that a single pyocin particle can kill a single bacterial cell [120] and can be used to target various bacterial pathogens and treat infections [121,122]. This is in contrast to T6SS where the killing of target cells has so far been associated only with functions of effectors that are being secreted by T6SS. This suggests that the Hcp tube is probably not stable and mere puncturing of the target cells by T6SS is not lethal. Indeed, unlike in phages or pyocins, the Hcp tube has not yet been detected as a stable structure ejected from a contracted sheath. It is likely that if the Hcp tube were stable, the T6SS+ cells would need a mechanism to specifically remove the Hcp tube from their cell envelope, for example by a specific cleavage, to allow quick sealing of the membranes to prevent their own death.

## 10. Concluding remarks

Many secretion systems have evolved to deliver proteins from bacterial cell cytosol to the extracellular space or across the target cell membrane. These systems vary in structure and mechanism of secretion and therefore also in the efficiency of translocation. Energetics of the T6SS is particularly interesting considering the fact that it seems as if only few molecules of spike-associated effectors are secreted with each contraction and since the T6SS seems relatively costly, considering the loss of Hcp and potentially large consumption of ATP during refolding of the contracted sheath. What are really the benefits of the T6SS mode of action? Why is T6SS used to secrete proteins? What are the main advantages of this mechanism of secretion?

One advantage could be that in the case of effectors that are preloaded into the Hcp tube, in one step, which can be accomplished in only a few tens of seconds, tens of various effectors could be delivered into the target cell at once. The second advantage is that a large amount of energy is potentially released during the contraction and this could be necessary for puncturing the target cell membranes. Delivery of folded hydrophilic proteins across membranes requires breakage of large amount of hydrophobic interactions between membrane lipids. This problem can be solved by an evolution of a transmembrane domain that inserts amphipathic segments into the membrane to create a hydrophilic conduit for the translocated protein. However, such a mechanism has to adapt to various membrane compositions and has potential limitations on the fold of the translocated protein. In the case of T6SS, the physical rupture of the target cell membrane might be enough to push large substrates across without a need for a protein dedicated to engage the membrane and potentially also without a limit to the structure of the translocated protein.

This might be the reason why membrane translocation by physical puncturing is conserved in many related systems: R-type pyocins and phages targeting bacteria [24], but also similar nanomachines targeting eukaryotes, such as antifeeding prophage [123], metamorphosis-associated contractile structures [124] or *Photorhabdus* virulence cassette [125]. There is no doubt that further studies of dynamics, structure and function of these fascinating nanomachines will help us to fully unravel their mode of action and unlock their potential use for cargo delivery.
